# Impact of the COVID-19 pandemic on women living with and beyond breast cancer: a qualitative study of women’s experiences and how they varied by social determinants of health

**DOI:** 10.1186/s12885-023-11351-x

**Published:** 2023-09-15

**Authors:** Charlotte Myers, Catherine Waldron, Kathleen Bennett, Caitriona Cahir

**Affiliations:** 1 School of Population Health, RCSI University of Medicine and Health Sciences, Dublin, Ireland; 2 Data Science Centre, School of Population Health, RCSI University of Medicine and Health Sciences, Dublin, Ireland

**Keywords:** COVID-19, Breast cancer, Social determinants of health, Quality of life, Health care disruption

## Abstract

**Background:**

The aim of this study is to explore the general impact of COVID-19 on the access and use of BC services and support and overall well-being in women living with a diagnosis of breast cancer (BC) and to investigate how these experiences varied by the social determinants of health (SDH).

**Methods:**

Semi-structured qualitative interviews were conducted with women selected through stratified purposive sampling to ensure data were available on information-rich cases. Interviews were conducted in early 2021 during government restrictions due to COVID-19. Thematic analysis was conducted to obtain overall experience and variation of experience based on SDH.

**Results:**

Thirty seven women participated in interviews. Three major themes, with additional subthemes, emerged from analysis: 1. breast cancer services (screening, active treatment, and routine care); 2. breast cancer support and communication (continuity of care, role of liaison, and support services); and 3. quality of life (QoL) and well-being (emotional well-being; social well-being; and functional well-being). Women’s experiences within the themes varied by socio-economic status (SES) and region of residence (urban/rural) specifically for BC services and support.

**Conclusion:**

The pandemic impacted women living with and beyond BC, but the impact has not been the same for all women. This study highlights areas for improvement in the context of BC care in Ireland and the findings will inform further policy and practice, including standardized BC services, improved communication, and enhancement of cancer support services.

**Supplementary Information:**

The online version contains supplementary material available at 10.1186/s12885-023-11351-x.

## Introduction

Cancer care during the COVID-19 pandemic was compromised globally [1], including disruptions to diagnosis, treatment, and routine care [2]. As a result, many cancer centres adapted management techniques and clinical guidelines to optimise treatment during the pandemic, such as priotizing high-risk patients, adjusting treatments to minimize hospital visits, and utilising telemedicine [3, 4]. Globally, breast cancer (BC) is one of the most common cancers in women; specifically in Ireland, BC accounts for one third of all major malignancies in women [5, 6]. Studies from varying countries, including Ireland, have indicated that women with a diagnosis of BC experienced disruptions to their BC care including delays, cancellations, and modifications [7]. Women with BC have also been physically, emotionally, and psychosocially challenged, resulting in reduced quality of life (QoL) [8, 9]. Specifically, women have reported worsened physical functioning and high rates of anxiety, depression, distress, and loneliness [10–12].

While research has shown a high impact of COVID-19 for women with BC, this impact may not be equal for all women. There are potential health inequalities pertaining to COVID-19 which highlight social and economic factors that influence an individual’s health [13]. To better understand health inequalities within the context of the COVID-19 pandemic, the social determinants of health (SDH) framework can be applied to describe the impact of social and health disparities on disease occurrence, distribution, and consequences [14]. During COVID-19, patterns of social disparities (e.g. socioeconomic status (SES), insurance status, education, and region) may be associated with greater disruption of health services, resulting in increased negative health outcomes and lower QoL [13, 15]. Prior to the pandemic, health inequalities in Ireland have been associated with income and health insurance status [16].

Few qualitative studies have been published [8, 17] and there is limited research on potential health inequalities pertaining to BC and COVID-19. Previous studies on health inequalities during COVID-19 have mainly been conducted in the USA and focus on ethnic disparities [18, 19]. Therefore, the aims of this qualitative study are to explore: (i) the general impact of COVID-19 on access and use of BC services and support and overall well-being in women living with and beyond BC in Ireland and (ii) how these experiences varied for women according to SDH.

## Methods

### Study design

The research methodology aligns with Consolidated criteria for Reporting Qualitative research (COREQ) reporting guidelines [20].

### Participants

The study sample included women who previously participated in a baseline survey of a prospective cohort study on the impact of COVID-19 on women with a diagnosis of BC (*N* = 387) [21]. All participants had a diagnosis of BC in the last 5 years, were living in Ireland, were aged 18 years of age or older, English speaking, and had no known serious psychiatric conditions. Participants completed the baseline surveys between September 2020 and April 2021 which corresponded with the second and third waves of COVID-19 infection and government restrictions in Ireland [22]. In total, 247 of women in the cohort (64%) provided consent to be contacted for an interview, and 63 women were invited to take part in the study via purposive sampling based on demographics (SDH) and time since diagnosis. Details on the purposive sampling strategy are in Supplementary 1. Ethical approval from the Office for National Research Ethics Committee in Ireland (20-NREC-COV-078).

### Procedure

Semi-structured, open-ended online interviews were conducted via Microsoft Teams (General Data Protection Regulation compliant) from April 2021 through May 2021 by two qualitatively-trained researchers (CM, CW) using a topic guide based on the preliminary findings of the survey study [21]. The topic guide included questions on the impact of COVID-19 on BC care and treatment, overall health and well-being, and coping skills and social support. The topic guide was piloted on 4 women; specific topics were added and deleted, and the wording was modified accordingly. Interviews were audio-recorded through Microsoft Teams and transcribed verbatim and anonymised.

### Data analysis

Thematic analysis was conducted using the following steps: familiarizing with data; generating initial codes; searching for themes; reviewing themes; defining and naming themes; and producing the report [23]. Further details on coding strategy are in Supplementary 2. The data were organised by themes and sub-themes (objective i) using simple coding queries. Cross-tabulation was used across the SDH, including SES and region, to address variation within themes and sub-themes (objective ii). Interpretation of analyses was conducted by the primary researcher (CM) to produce thematic results. Illustrative quotes have been provided to supplement narrative descriptions.

## Results

### Participant characteristics

Supplementary 3 displays the flowchart for study recruitment. Forty-two women participated in interviews (67% response rate), and 37 of the interviews were included in the final analysis (4 pilot interviews, 1 excluded due to software recording error). Supplementary 4 displays the SDH and clinical characteristics of the women invited and included in the study via purposive sampling strategy and Supplementary 5 displays further details of the women included in the study.

### Main themes and sub-themes

The following main themes were identified from the analysis of the impact of COVID-19 on women with BC in Ireland: 1. breast cancer services and treatment; 2. breast cancer support and communication; 3. QoL and well-being. Supplementary 6 illustrates each theme with embedded sub-themes. Each theme will first be presented to describe the general experience of COVID-19 on women with BC (objective i) and then the differences within themes and sub-themes are identified across the SDH, including SES and region (objective ii).

#### Breast cancer services and treatments

Cited quotations can be found in Table 1.

**Table 1:** Quotes on overall experiences and variation of experience based on SDH for women receiving BC services during COVID-19

	**Diagnostic/ screening**	**Active treatment**	**Routine care**
**Overall experiences**	*“I felt a lump… so I went to my own GP. She saw me and she faxed a letter straight into [cancer centre]. Within a week, I was in [cancer centre] having seen the doctors and then followed on with biopsies and mammograms.”* -P16- (50–64 years, diagnosed in 2020, mid-SES, urban)	*“Well, it was a stressful period of time because I felt it was growing. And I couldn’t do anything about it. And the hospitals weren’t really taking patients… But eventually I got called again into the… clinic. I had my operation there.”* -P8- (> 65 years, diagnosed in 2020, high-SES, urban)	*“…the phone calls are very handy for somebody who lives a good distance from the hospital. You know, if it is something that is doable over the phone, that’s great.”* -P34- (≤ 49 years, diagnosed 1–2 years prior, low-SES, rural)
		*“I do wonder was it because it was a phone consultation that information was missed when I was moving from the breast clinic to oncology.”* -P14- (≤ 49 years, diagnosed in 2020, mid-SES, urban)	*“…the oncology appointment was over the telephone. So, I didn’t find that very satisfactory. It’s just, normally, I like to have my breast checked by a professional. Even though I check myself and my history isn’t of lumps or anything, but it’s just reassuring.”* -P27- (50–64 years, diagnosed 1–2 years prior, low-SES, urban)
**Variation of experience**	*“What happened was… I got an appointment sent out for October. And I phoned them and they said to me look, there’s probably other women that met the criteria, that needed the mammogram before me.”* -P25- (≤ 49 years, diagnosed in 2020, low-SES, urban)	*“When the chemo was finished, I had an appointment…to meet the surgeon to decide on my surgery…I got a letter telling me I had the appointment and then I got a text message saying it was cancelled.”* -P32- (≤ 49 years, diagnosed in 2020, low-SES, urban)	*“I haven’t had an MRI in about a year and I meant to be getting them every six months. So then when I did, [the lump] just showed up.”* -P35- (≤ 49 years, diagnosed 3–5 years prior, low-SES, rural)
		*“I’d wait for three months or such time until COVID was over… before he would do anything. And I, at that time, I just said “no, I can’t wait that long, that’s too long.”* -P12- (> 65 years, diagnosed in 2020, high-SES, rural)	*“And they were going to refer me for, I think, one or two scans. Obviously, I’ve heard nothing about that because of lockdown. And then my mammogram last year was cancelled.”* -P23- (50–64 years, diagnosed 3–5 years prior, mid-SES, rural)

##### Overall experience (objective i)

Many appointments disruptions included cancellations, delays, and/or modifications, including telemedicine. Despite these disruptions, the majority of women expressed high regard for their BC care team and many women acknowledged their appointments, when seen in-person, to be more organized and more hygienic during the pandemic. Sub-themes align with the type of breast cancer service: diagnostic/ screening; active treatment (e.g. surgery, chemotherapy, and/or radiation therapy); and routine care (e.g. follow-up appointments, exams, scans, and other tests). In terms of the sub-theme diagnostic/screening, the majority of women interviewed were diagnosed prior to the pandemic; of the women who did receive a diagnosis (*n* = 6) during the pandemic, most were referred promptly (P16).

Cancellation of active treatment appointments was not common. Moreso, women experienced delayed or modified appointments and services for their active treatment. For example, appointments to schedule specific treatments, such as surgery, were postponed which resulted in not only delayed treatment, but also health-related stress (P8). Similarly, modified appointments, such as telemedicine, caused women distress about their BC prognosis during active treatment (P14). However, other women experienced positive modifications such as expedited surgery and radiotherapy and relocation of chemotherapy sessions.

Routine care services were the most frequently modified appointment during COVID-19. Women who transitioned from active treatment to post-treatment during the pandemic year found it to be unsettling without in-person visits to their BC care team. Women who completed their active treatment around onset of the pandemic felt they were lost in the system immediately following specific treatments, such as surgery. Routine appointments were modified to be virtual and several women expressed appreciation for the convenience of virtual consultations when seeing a consultant face-to-face was not necessary (P34), yet other women expressed apprehension and dissatisfaction in virtual appointments for practical reasons, such as manual breast examinations (P27).

##### Variation of experience based on SDH (objective ii)

Many women were aware of the prioritization for certain high-risk cases, but they did not understand how needs were assessed for diagnostic pathways, active treatment, and routine services. For diagnostic services, most women who were diagnosed during the pandemic received adequate, timely diagnostic services. The referral process and diagnosis was delayed for one woman of low-SES (P25).

For the women receiving active treatment during the pandemic, there were evident variations in disruption to services according to SDH. More extreme disruptions, such as cancelled appointments or postponed treatments, were only evident for women of lower-SES (P32). Furthermore, one woman of low-SES felt rushed to leave the hospital following surgery; at the time of being interviewed, she was still waiting for her radiotherapy to be scheduled. There was also a lack of available treatment for one woman living in a rural setting during COVID-19 (P12).

There was also variation in disruptions in routine care according to SDH. Major disruptions in routine services were more common for women of lower-SES. Specific routine services, such as scans and blood tests, need to be conducted in-person and women of low-SES expressed adverse events resulting from inadequate routine follow-up care, such as scans and blood tests (P35). Additionally, women of lower-SES, especially those living rurally, expressed more concern for postponed and cancelled appointments, as such disruptions often lead to delayed or absent test results (P23).

#### Breast cancer support and communication

Cited quotations can be found in Table 2.

**Table 2:** Quotes of overall experiences and variation of experience based on SDH for women with their BC support and communication

	**Continuity of care**	**Role of Liaison**	**Support services**
**Overall experiences**	*“When the hospitals merged, nobody knew what was happening. The nurses didn’t know, the doctors didn’t, it was all just up in the air, they were terrified, no one knew anything. And even getting all my scans, I was chasing them. They didn’t know whether they were happening in public, were they happening in private…* *Who was doing them? That was very stressful.”* -P21- (≤ 49 years, diagnosed in 2020, mid-SES, rural)	*“…any questions I had, I had access to [my] breast care nurse, anytime I needed to talk to somebody and you know, she was there on hand to answer any queries I had or worries I had.”* -P20- (≤ 49 years, diagnosed in 2020, mid-SES, rural)	“*I do feel if the massage was there when I was going through the chemo… that I would have attended them. But it wasn’t there so you couldn’t, and I’m suffering now from it, which maybe if [the] services [were] there, I wouldn’t need the physio now and I wouldn’t be in as much pain as I am in at the minute with my muscles and shoulder.”* -P32- (≤ 49 years, diagnosed in 2020, low-SES, urban)
	*“And the oncology ward moved outside to an outdoor building, which was great, so you were just going in and it was all oncology patients and oncology nurses. That was it. You didn’t have any contact with anyone else or anything like that, which was great.”* -P32- (≤ 49 years, diagnosed in 2020, low-SES, urban)	*“There isn’t any link between chemo, radiotherapy, and then finishing. Like, there isn’t a liaison nurse… there was a nurse I could talk to in oncology, but it depended on the day. So…there needs to be someone that will act as a liaison for you. And that there has to be some continuance afterwards…not just that they drop you.”* -P1- (≤ 49 years, diagnosed 3–5 years prior, high-SES, urban)	“*Well, I had virtual physiotherapy after the mastectomy… [and] to be honest with you, that was probably the one thing that I felt that you really shouldn’t have done virtually. Like, under my arm still is quite numb, even you know, all this time later.”* -P25- (≤ 49 years, diagnosed in 2020, low-SES, urban)
**Variation of experience**	*“…hearing from different members of his team. And getting different answers for my questions. It was just, it was awful. It was so frustrating and so upsetting. Again not feeling like I was being properly treated…”* -P26- (≤ 49 years, diagnosed 1–2 years prior, low-SES, urban)	*“I wouldn’t really have a direct contact for any particular person. Like I would say… I don’t actually know who I would contact. I don’t know.”* -P37- (50–64 years, diagnosed 1–2 years prior, low-SES, rural)	*“I have had no access to the likes of physiotherapy. I had [a] quartered nerve after surgery that usually is treated by physiotherapy. I have not been able to do that..”* -P34 - (≤ 49 years, diagnosed 1–2 years prior, low-SES, rural)

##### Overall experience (objective i)

Women emphasized the importance of support and communication across the BC care continuum. Specifically, women mentioned the following sub-themes: continuity of care; role of a liaison; and BC support services. There was a general emphasis on the importance for continuity of care for BC, yet women expressed poor continuity of care during the pandemic. A lack in continuity of care was distressing, especially during the transition out of active treatment. The lack of continuity could be the result of inadequate collaboration and varying treatment locations. For example, private clinics were used by public hospitals for treatment and some women who typically receive their cancer treatment publicly were seen in private clinics (P21). However, the change in location allowed for safe and effective treatment during the pandemic (P32).

To ensure continuity of care, most women described the importance of immediate contact with a nurse or individual within their BC care team. Women described this main contact as a liaison, someone who could mediate BC services with them, across all stages of the cancer continuum. Direct contact with this nurse provided women confidence (P20) and enhanced cohesion in their cancer care. The majority of women expressed the assignment of a main nurse during specific treatments (e.g. chemotherapy) but there was a lack of cohesion between treatments and when transitioning from active treatment (P1).

BC support services (e.g. counselling, physiotherapy, lymphedema therapy) were described as beneficial and women who took advantage of such resources greatly benefitted from them, however, others were not made aware of the available resources. Women diagnosed during the pandemic experienced more disruptions in support services, which affected overall well-being (P32). Women found virtual counselling and support groups during COVID-19 to be beneficial, however women found virtual physiotherapy appointments to be of little use (P25).

##### Variation of experience based on SDH (objective ii)

Experience of BC support and communication varied by SES. Women of lower SES expressed worse continuity of care compared to women of higher SES. This distinction appeared to be exacerbated for women who received care in multiple locations. While some women of high-SES who received cancer treatments in varying locations found their experience to be cohesive, other women of low-SES found their experience lacked continuity. For example, one woman expressed the confusion and miscommunication between her BC care providers (P26); other women experienced miscommunication with their BC clinic which caused missed appointments. To enhance overall cohesion in BC care, several women suggested proper linkage between treatment locations and when transitioning to survivorship. Proper linkage may include contact with a liaison nurse. As a result of living in a rural area, one woman of low-SES described attending different clinics for varying treatments as stressful without a liaison to help navigate her BC journey (P37).

Furthermore, there were inconsistencies in closures for support services. Some women of high-SES were able to attend physiotherapy during the pandemic, yet one woman of low-SES was unable to access such services, a disruption still impacting her physical functioning (P34).

#### Well-being and quality of life (QoL)

Cited quotations can be found in Table 3.

**Table 3:** Quotes of overall experiences and variation of experience based on SDH for women and their QoL

	**Emotional well-being**	**Social well-being**	**Functional well-being**
**Overall experiences**	*“I couldn’t let that fear stop me. I mean, I had the fear, but cancer doesn’t wait for anyone to catch up. So I said, alright…the fear of cancer is worse than the fear of COVID. I can take steps to protect myself.”* -P20- (≤ 49 years, diagnosed in 2020, mid-SES, rural)	*“And you just have to go in on your own and you know [my sister] couldn’t even sit in the waiting room with me… I just knew then there was a pandemic on. Everything was just like… you’re on your own. From this point on, you know which I understood. But it was so hard, so hard.”* -P36- (50–64 years of age, diagnosed in 2020, low-SES, rural)	*“I used to be so fit, I used to run every day on my lunch break…And I just found during the pandemic that, you know, you’re limited to two kilometres, then it was five, and it’s just got to be so boring.”* -P23- (50–64 years of age, diagnosed 3–5 years prior, rural, mid-SES)
	*“It was a stressful period of time because I felt [the cancer] was growing. And I couldn’t do anything about it. And the hospitals weren’t really taking patients, so it was a stressful time for everybody.”* -P8- (> 65 years, diagnosed in 2020, high-SES, urban)	*I was a bit shocked to be diagnosed with breast cancer and I was more shocked when it came down with the pandemic. I couldn’t really do anything about it. And also the fact that I couldn’t see any of my family, so there was nobody really… so isolation is probably the biggest thing.”* -P8- (> 65 years, diagnosed in 2020, high-SES, urban)	*“Last year was very difficult in lots of ways because I only finished my treatment [and I] had a lot of fallout… from the treatment gradually over the year. I struggled. I had an awful lot of pain.”* -P5- (50–64 years of age, diagnosed 1–2 years prior, high-SES, urban)
**Variation of experience**			*“[The] financial implications of being off for a year and a half…it puts a hold on everything. You know, we wanted to try for another child. But we couldn’t because I need to get my finances in order again before we were to go down that route. So it’s delayed, it’s put a delay on everything in life* -P26- (≤ 49 years, diagnosed 1–2 years prior, low-SES, urban)

##### Overall experience (objective i)

QoL was another common theme, including emotional, social, and functional well-being. While the pandemic has impacted the emotional well-being of the general population, women with BC experienced specific challenges. Women expressed heighted anxiety during COVID-19, which can be attributed to contracting COVID-19 and/or worsened cancer prognosis. Women explained how mentally difficult it was for them to balance being safe and feeling secure with their cancer (P20). As a result, the fear of cancer recurrence was common for most women interviewed for the study. While this fear is present during normal circumstances, the pandemic exacerbated it with the lack of medical contact (P8).

Most women felt lonely and isolated during COVID-19, impacting their social well-being. Those women who received treatment during the pandemic expressed loneliness in the hospital or clinic setting due to strict limitations to visitors; they were unable to bring someone along with them for support (P36). Furthermore, women found it difficult to minimize socialization with family and friends during the pandemic due to social isolation guidelines, which impacted their ability to cope (P8).

Regarding functional well-being some women increased their physical activity during the pandemic due to a more flexible daily routine during the pandemic. However, other women experienced worsened physical activity due to strict government restrictions and access to resources (P23). The closure of BC services, such as physiotherapy, impacted women’s physical functioning and pain, especially for women with a recent diagnosis of BC, who were still experiencing side effects from BC treatment (P5). Women also addressed a change in their daily life; women who were employed prior to the pandemic adjusted to remote working. While some women expressed negative aspects to working from home, other women experienced an improvement to their daily life by avoiding travel time due to commuting. However, women who continued to work outside their home faced excess stress and fear related to COVID-19 exposure.

##### Variation of experience based on SDH (objective ii)

There was no variation of experience based on SDH for the sub-themes emotional and social well-being, however, there were differences in functional well-being. The majority of women did not experience a detrimental change to their financial stability during the pandemic, however women of lower-SES experienced more financial difficulties (P26).

## Discussion

This study used qualitative methods to explore the experiences of the COVID-19 pandemic on women living with a diagnosis of BC and whether these experiences varied by SDH, including SES and region. Most women experienced disrupted BC services across the cancer continuum, which is consistent with the international literature, along with research specific to Ireland [21, 24]. Given that many BC facilities operated at a reduced capacity during COVID-19 [25], most women who were diagnosed around the onset of the lockdown were given priority for triage into cancer centres, which indicates an adequate pathway regardless of screening programme closures. To compensate for the reduction of in-person visits, telemedicine was used as a common modification to BC services [26]. Telemedicine can eliminate barriers such as distance to BC clinic, transportation, and cost [27] and it can improve the communication between patient and BC care team [28].

Prior to the pandemic, previous research in Ireland found that women undergoing treatment for BC lacked coordination between treatments and transitioning out of active treatment. For example, women who were assigned a liaison nurse during surgical care lost communication when transitioning to oncology [29]. The pandemic resulted in more modifications to BC services, which resulted in poorer communication and cohesion. Most women suggested the appointment of a liaison nurse to oversee all treatments and all phases across the BC care continuum, which has been found to be beneficial for continuity [30].

Women’s concerns evolve along the BC care continuum [31], and the reduction of BC support services and resources (e.g. such as physiotherapy and counselling) during the pandemic impacted women’s QoL, including physical and emotional health, which is consistent with the current literature [9, 21]. Many women expressed vulnerability, anxiety, fear of cancer recurrence, and loneliness during the pandemic, which are psychosocial concerns reported previously in the literature for women with BC [32, 33]. However, the impact of the pandemic has had significant impact on women’s emotional well-being and it may vary based on treatment status [8], which is consistent with findings in our study.

This study also found that experiences varied according to SDH, including SES and region. Women with lower-SES experienced greater disruption to BC care during the pandemic, which is consistent with research conducted in other countries [28, 34]. Literature has identified the ability to pay (i.e. SES) as an independent risk factor to BC care disruption during COVID-19 [35], along with region [36]. Ireland remains the only western European country that does not provide universal primary health care to all citizens [37] which creates major health inequities based on the ability to pay rather than clinical need [38]. During the pandemic, health insurance was found to be a significant SDH for BC service disruption and QoL specific to BC [21]. The current study corroborates such results and indicates that the COVID-19 pandemic has further exacerbated health inequities in Ireland [39].

Women described insufficient communication with their BC care team, which corroborates with existing literature [40]. The assignment of a liaison nurse can improve communication and collaboration among all aspects of multidisciplinary care, and in turn improving psychosocial outcomes for the individual [30]. However, there were varying experiences with an appointed nurse based on region and SES, which may indicate a disparity in the coordination of BC care. There were no SDH which impacted women’s emotional well-being, however, research prior to the pandemic identified the following predictors of distress among BC survivors: age; ethnicity; SES; and marital status [31]. In our study, low-SES was associated with more financial distress, which is similar with research conducted in the USA which found that public health recipients experienced more financial difficulties during COVID-19 [18].

This qualitative study included a large range of participants who were selected via stratified purposive sampling to ensure representative of women living with and beyond BC. The interviews were timely and represent the immediate and diverse experiences of the pandemic, which provides knowledge on a topic with few published qualitative studies. However, the study was unable to completely capture the impact of COVID-19 on screening services, as participants who enrolled in the study already had a diagnosis of BC.

## Conclusion

In conclusion, this study depicts the interrelationship between BC and the broader context of SDH during the COVID-19 pandemic. Women living with and beyond BC experienced disruptions to their BC services across the cancer care continuum, reflecting the vulnerability of healthcare systems during unprecedented challenges. The prioritization of cancer centre triage and the integration of telemedicine underscore the adaptability of healthcare systems to maintain essential BC services, despite the suspension of BC screening programmes. However, the disruptions to BC services have magnified pre-existing issues within the healthcare system in Ireland, particularly in relation to communication and care continuity. Importantly, the pandemic has also highlighted the emotional challenges along the BC care continuum, reflecting heightened anxieties, persistent fears of cancer recurrence, and a sense of isolation. Moreover, the study demonstrates that these impacts are not uniform, with distinct disparities influenced by SES. Low-SES individuals experience intensified disruptions and financial distress, mirroring wider socio-economic inequalities exacerbated by the pandemic. The study accentuates the crucial role of SES as a determinant of access to care and psychosocial well-being, highlighting the need to address systemic inequities. As we navigate the post-pandemic era, the insights from this research emphasize the necessity of a standardized approach to BC care, fostering comprehensive services and enhanced communication to alleviate the psychosocial burdens faced by those dealing with breast cancer, thereby prioritizing QoL within the context of SES.

### Supplementary Information

**Additional file 1: Supplementary 1.** Purposive sampling strategy. **Supplementary 2.** Coding Strategy. **Supplementary 3.** Flowchart for recruitment from the survey study to enrollment for the interview study. **Supplementary 4.** Demographic and clinical strata for women invited and enrolled in the study. **Supplementary 5.** Demographic and clinical characteristics of the women with BC interviewed for the study (*n* = 37). **Supplementary 6.** Concept map with main themes and subthemes.

## Data Availability

The datasets generated and/or analysed during the current study are not publicly available due the privacy of individuals that participated in the study, but are available from the corresponding author on reasonable request. Information as can be shared by additional summary level data without individual data.

## Abbreviations

BC: Breast cancer
SDH: Social determinants of health
QoL: Quality of life
SES: Socio-economic status
