# Supplementary material for: Impact of the COVID-19 pandemic on women living with and beyond breast cancer: a qualitative study of women’s experiences and how they varied by social determinants of health
Source: BMC Cancer. 2023 Sep 15;23:867. doi: 10.1186/s12885-023-11351-x (PMC10503161; doi:10.1186/s12885-023-11351-x)

Supplementary 1

Purposive sampling strategy:

For recruitment, women were categorized into strata by SDH including age, region, health insurance status, and education, and also by clinical characteristics including time since cancer diagnosis. Women were purposively sampled within these strata to ascertain any variation in experiences of COVID-19; 63 women were invited to take part in the study. After recruitment, the SDH and clinical characteristics were further refined into the following strata: age; region; socio-economic status (SES); and time since diagnosis. For age, women were categorised as; ≤49 years, 50-65 years, ≥65 years. For region, participants were categorised as urban (living in a city/ town) vs. rural (living in a village/ countryside). SES was established by combining level of education and health insurance status. Women with low education and no private health insurance were determined to have low-SES; women with either low education and private health insurance or high education and no private health insurance, taking into consideration age and working status were determined to have mid-SES; women with high education and private health insurance were determined to have high-SES. For the clinical characteristic time since diagnosis, participants were categorised as diagnosed within 2020, diagnosed 1-2 years prior to the pandemic, or diagnosed 3-5 years prior to the pandemic.

Supplementary 2

Coding Strategy:

Initially, two researchers (CM, CW) independently coded two interviews and compared and discussed results to develop a coding index. The coding index was achieved by identifying recurrent themes and subthemes introduced into the interviews through the topic guide. Once the codebook was agreed upon, both researchers then independently coded another four interviews; the codes from the four interviews were then compared using NVivo 12 (QSR International) and inter-coder reliability (ICR) was calculated. Percentage agreement is an adequate measure of ICR for nominal variables and a percentage agreement within 10% was found for each code. Once ICR was ensured, the primary researcher (CM) used the codebook to code all interview transcripts in NVivo 12. The codebook was applied deductively to the data and it was also modified inductively during the coding process to reflect the content of the data, as needed.

Supplementary 3

Flowchart for recruitment from the survey study to enrollment for the interview study


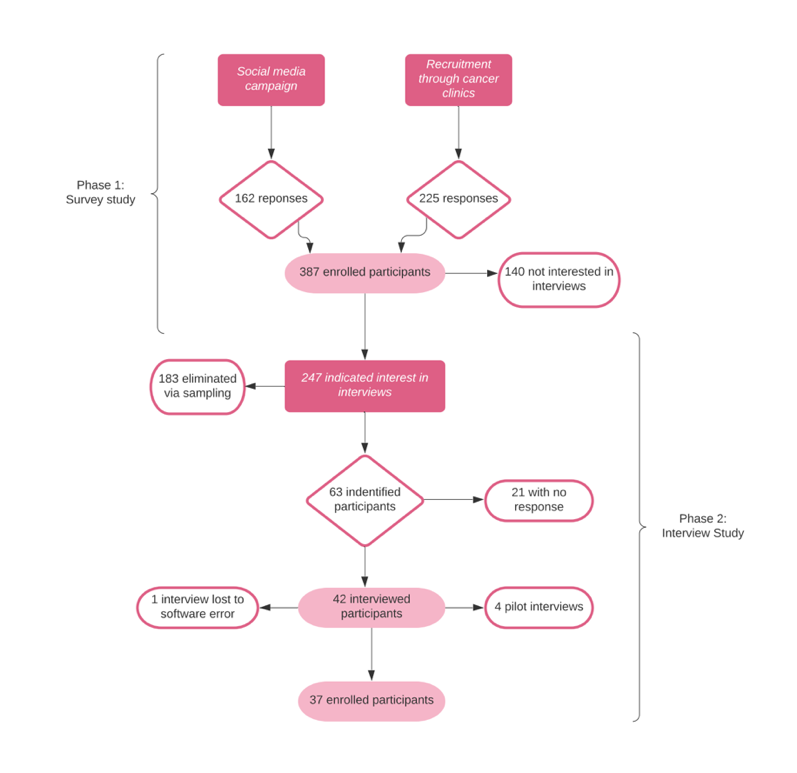


Supplementary 4

| Demographic and clinical strata for women invited and enrolled in the study | | | |
| --- | --- | --- | --- |
| Strata | Sub-group | Invited (N=63)  N (%) | Enrolled (N=37)  N (%) |
| I. Age | 49 years or younger | 21 (33.3) | 16 (43.2) |
|  | 50 to 64 years of age | 20 (31.7) | 10 (27.0) |
|  | 65 years or older | 22 (34.9) | 11 (29.7) |
| II. Time since diagnosis | Diagnosed within 2020 | 21 (33.3) | 15 (40.5) |
|  | Diagnosed 1-2 years prior | 21 (33.3) | 11 (29.7) |
|  | Diagnosed 3-5 years prior | 21 (33.3) | 11 (29.7) |
| III. SES | High | 20 (31.7) | 13 (35.1) |
|  | Mid | 21 (33.3) | 11 (29.7) |
|  | Low | 22 (34.9) | 13 (35.1)­­ |
| IV. Region | Urban | 36 (57.1) | 22 (59.5) |
|  | Rural | 27 (42.9) | 15 (40.5) |

Supplementary 5

Demographic and clinical characteristics of the women with BC interviewed for the study (n=37)

| ID | Socio-economic status | Region | Age | Diagnosis |
| --- | --- | --- | --- | --- |
| P1 | High | Urban | 49 years or younger | Diagnosed 1-2 years prior |
| P2 | High | Urban | 49 years or younger | Diagnosed 3-5 years prior |
| P3 | High | Urban | 49 years or younger | Diagnosed 3-5 years prior |
| P4 | High | Urban | 50 to 65 years of age | Diagnosed within 2020 |
| P5 | High | Urban | 50 to 65 years of age | Diagnosed 1-2 years prior |
| P6 | High | Urban | Older than 65 | Diagnosed within 2020 |
| P7 | High | Urban | Older than 65 | Diagnosed within 2020 |
| P8 | High | Urban | Older than 65 | Diagnosed within 2020 |
| P9 | High | Urban | Older than 65 | Diagnosed 1-2 years prior |
| P10 | High | Urban | Older than 65 | Diagnosed 1-2 years prior |
| P11 | High | Rural | 49 years or younger | Diagnosed 3-5 years prior |
| P12 | High | Rural | Older than 65 | Diagnosed within 2020 |
| P13 | High | Rural | Older than 65 | Diagnosed 1-2 years prior |
| P14 | Mid | Urban | 49 years or younger | Diagnosed within 2020 |
| P15 | Mid | Urban | 49 years or younger | Diagnosed 1-2 years prior |
| P16 | Mid | Urban | 50 to 65 years of age | Diagnosed within 2020 |
| P17 | Mid | Urban | 50 to 65 years of age | Diagnosed 1-2 years prior |
| P18 | Mid | Urban | Older than 65 | Diagnosed within 2020 |
| P19 | Mid | Urban | Older than 65 | Diagnosed 3-5 years prior |
| P20 | Mid | Rural | 49 years or younger | Diagnosed within 2020 |
| P21 | Mid | Rural | 49 years or younger | Diagnosed within 2020 |
| P22 | Mid | Rural | 49 years or younger | Diagnosed 1-2 years prior |
| P23 | Mid | Rural | 50 to 65 years of age | Diagnosed 3-5 years prior |
| P24 | Mid | Rural | 50 to 65 years of age | Diagnosed 3-5 years prior |
| P25 | Low | Urban | 49 years or younger | Diagnosed within 2020 |
| P26 | Low | Urban | 49 years or younger | Diagnosed 1-2 years prior |
| P27 | Low | Urban | 50 to 65 years of age | Diagnosed 3-5 years prior |
| P28 | Low | Urban | 50 to 65 years of age | Diagnosed 3-5 years prior |
| P29 | Low | Urban | Older than 65 | Diagnosed 3-5 years prior |
| P30 | Low | Urban | Older than 65 | Diagnosed 3-5 years prior |
| P31 | Low | Rural | 49 years or younger | Diagnosed within 2020 |
| P32 | Low | Rural | 49 years or younger | Diagnosed within 2020 |
| P33 | Low | Rural | 49 years or younger | Diagnosed within 2020 |
| P34 | Low | Rural | 49 years or younger | Diagnosed 1-2 years prior |
| P35 | Low | Rural | 49 years or younger | Diagnosed 3-5 years prior |
| P36 | Low | Rural | 50 to 65 years of age | Diagnosed within 2020 |
| P37 | Low | Rural | 50 to 65 years of age | Diagnosed 1-2 years prior |

Supplementary 6

Concept map with main themes and subthemes


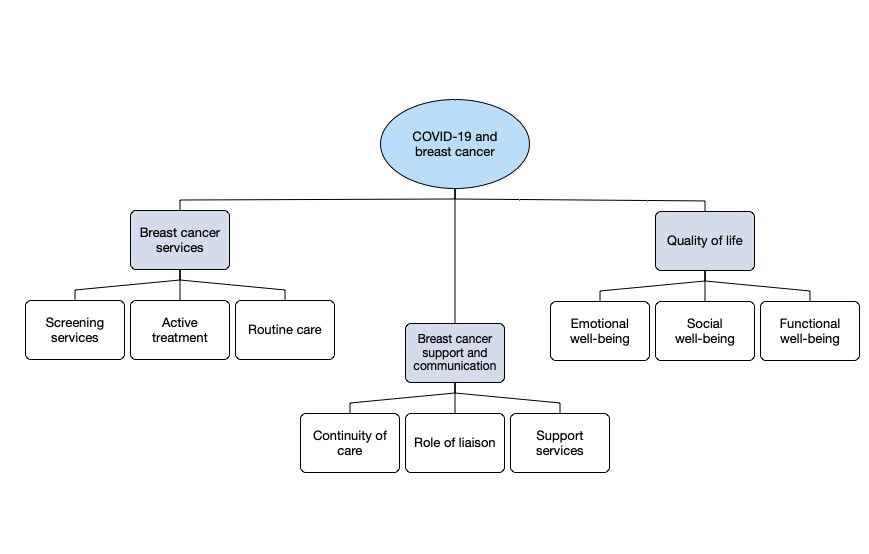

Supplement: Supplementary file 1 — Additional file 1: Supplementary 1. Purposive sampling strategy. Supplementary 2. Coding Strategy. Supplementary 3. Flowchart for recruitment from the survey study to enrollment for the interview study. Supplementary 4. Demographic and clinical strata for women invited and enrolled in the study. Supplementary 5. Demographic and clinical characteristics of the women with BC interviewed for the study (n = 37). Supplementary 6. Concept map with main themes and subthemes. [file 12885_2023_11351_MOESM1_ESM.docx]
